# Weight of the Lungs

**Published:** 1864-10

**Authors:** H. Allen

**Affiliations:** Asst. Surg. U. S. A.


### WEIGHT OF THE LUNGS.

By H. ALLEN, M. D., Asst. Surg. U. S. A.

In a series of post-mortem examinations, 200 in number, made at the Lincoln General Hospital, Washington, D. C., during the past eighteen months, the weight of the various organs was carefully noted. It has since been suggested that the conclusions drawn from the weighing of the lungs in these cases be recorded, since anatomists have paid but little or no attention to the subject.

Out of the 200 pairs of lungs 68 were found healthy. The smallest weight of any of this number was observed in a case of chronic diarrhœa; the right lung weighing but 8½ oz., the left 9 oz. The largest weight was seen in a case of diphtheria the right weighing 20 oz., the left lung 18½ oz. The average weight of the whole number was, for the right lung, 15⅓ oz., for the left lung, 14½ oz. In 7 out of the 41 cases of chronic diarrhœa, the lungs gave small weight. The aspect of the organ in these cases was peculiar. They were shrunken, and occupied but a small portion of the thoracic cavity; the distance between the anterior thoracic wall and the lung being in some specimens as great as 2½ inches. The parenchyma was dry, with little bronchial secretion evident, and no appearance of the pinkish hue of the healthy lung was presented. Owing to the absence of blood and the presence of pigmentary matter, the tissue was everywhere of a greyish color. The remaining 132 specimens were diseased; the majority from pneumonia and pleurisy. A large number were congested and rendered heavy, the parenchyma being charged either with the sero-sanguineous fluid of commencing pneumonia, or the greyish œdema of imperfect convalescence from the same disease. It would be uninstructive to give the weight of the lungs in each case, since no two specimens were alike; they varied greatly in the degree of complication of the congestion or consolidation. The heaviest organ was observed in a case of nearly complete double pneumonia; in which the last stage of red hepatization was attained in both lungs, with the exception of the apex of first lobe, left side, which crepitated under pressure. The right lung weighed 50 oz., the left 52. The smallest weight of any diseased lung was noticed in a case of phthisis, in which the relative weight of right and left lungs was as 8½ oz. to 7½ oz. The first 75 of these weights were recorded under the auspices of Assistant Surgeon G. M. McGill, U.S.A., the remainder by myself. The examinations were all made shortly after death, and it is believed that no decomposition had taken place in any of the specimens.—*Am. Med. Times.*

*Foreign Bodies in the Ear.*—For the removal of foreign bodies we should first employ only the gentlest means, such as syringing the ear with warm water; and by this, substances of the most different form and composition—even lead pencil—may be removed. Beyond a bent forceps, an ear-scoop with a long handle, and a small corkscrew, almost all the instruments recommended for this purpose are more or less toys or dangerous. By means of the corkscrew, wadding and similar soft substances may be easily drawn out; and in many cases we can remove bodies by passing the ear-scoop behind them. We should never employ force, and never should pass any instrument a line further into the meatus than we can follow it with the eye. For want of such a precaution many a patient has lost his life or his hearing. The first effect of rough procedures is to make matters more obscure, the bleeding and swelling which ensue rendering complete inspection impossible If the gentlest endeavors (or syringing), during which the eye guides the hand, do not succeed, the body should be left in the ear—aye, even were it a dagger's point; and strong as the expression seems, the author justifies it be reference to cases on record in which pointed bodies have remained for years in the ear with impunity. It is not meant to be said that bodies should in general be left in the ear, but that matters should not be made worse than they are by violent manipulations. Leaving the body in the ear, then, warm-water syringing and soft poultices are to be daily resorted to, until the ensuing suppuration loosens it and gives it a new direction. —*Dublin Med. Press*, Jan., 1864.

